# Nutritional status of under-five children born to teenage mothers in an urban setting, south-western Nigeria

**DOI:** 10.1186/s13104-019-4147-x

**Published:** 2019-03-04

**Authors:** Monday Daniel Olodu, Adewunmi Grace Adeyemi, Samuel Anu Olowookere, Olapeju Adefunke Esimai

**Affiliations:** Community Health Department, Obafemi Awolowo University, Ile-Ife, Osun State Nigeria

**Keywords:** Nutritional status, Teenage mothers, Breastfeeding practice, Under-fives

## Abstract

**Objective:**

There have been many studies on the nutritional status of under-fives and factors responsible but very few looks at this special group of women. This study assessed the breastfeeding practices of teenage mothers and determined its association with the nutritional indices of their under-five children. The study was a descriptive cross-sectional survey. A total of 300 mother–child pair was selected using a multi-stage sampling technique from Primary Health Care centres in Ondo West Local Government Area, Ondo State, Nigeria. Bivariate and multivariate logistic regression were done to identify predictors’ of poor nutritional status at p < 0.05.

**Results:**

About 87% initiated breastfeeding less than 1 h after birth while 31.9% breastfed their children exclusively for 6 months. Prevalence of stunting, wasting and underweight among the under-fives were 18.6%, 25.3%, and 29.5% respectively. Initiation of breastfeeding more than 1 h after birth increased the odds of stunting (OR = 9.551, CI = 1.279–16.310) and underweight (OR = 6.674, CI = 3.159–14.097) by about 10 and 7 times respectively. Whereas odds of wasting (OR = 2.346, CI = 1.228–4.480) was 2 times higher with breastfeeding duration less than 6 months. Therefore, education of teenage mothers on breastfeeding initiation and duration is vital in reducing malnutrition among under-fives.

## Introduction

Malnutrition is a teething public health problem affecting mostly the under-five children living in developing countries [1]. The 2013 Nigeria Demographic and Health Survey (NDHS) [2], revealed that 37% of children under the age of five are stunted, while 21% are severely stunted, eighteen percent of under-five children in Nigeria are considered wasted and 9% are severely wasted while 29% are underweight, with 12% being severely underweight. The under-five mortality rate in Nigeria is 128/1000 live birth which is among the world’s highest [2].

Child care is one of the key underlying causes of childhood malnutrition. Child care is manifested in the way a child is fed, nurtured, socialized and guided. It is practised by women who carry out some of the care activities such as breastfeeding and feeding of young children; psychosocial stimulation of children and support for their development; complementary food preparation, feeding and storage practices; hygiene practices and care for a child during illness and adoption of health seeking practices [3]. The complexity of child care requires that every woman must be adequately prepared to succeed in the responsibilities. However, every year, an estimated 21 million girls aged 15 to 19 years, and 2 million girls aged under 15 years become pregnant in developing countries [4, 5]. Evidence has shown that teenage pregnancy in Nigeria does not only account for high birth rate for teenagers, but that the incidence of pregnancy among female teenagers in Nigeria is increasing rapidly. In fact, the 2013 NDHS [2] showed that 23% of women aged 15–19 years have already begun childbearing.

Studies have shown that the high rate of teenage pregnancy contributes significantly to the high prevalence of malnutrition among under-five children [6, 7]. Maternal factors such as age, education, family size and marital status have also been found to significantly influence the nutritional status of their under-five children [8, 9]. Poor breastfeeding and complementary feeding practices also play key roles in determining the nutritional status of under-five children. In Nigeria, only 17% of children below 6 months are exclusively breastfed while 10% of children aged 6–23 months are fed appropriately based on recommended infant and young child feeding (IYCF) practices [2]. Poor nutritional status, especially among children, have negative effects on their health and development during the early years of life [10, 11].

There have been many studies on nutritional status of under-five children and factors responsible but very few looks at this special group of women, their breastfeeding practice and its influence on the nutritional status of their under-fives. This study, therefore, assessed the breastfeeding practices of teenage mothers, nutritional status of their under-five children and determined the association between breastfeeding practice and nutritional indices of children born to teenage mothers in an urban setting, south-western, Nigeria.

## Main text

### Methods

#### Setting and design

This facility based descriptive cross-sectional study was conducted at Primary Health Care (PHCs) centres, Ondo West Local Government Area (LGA), Ondo State, Nigeria from February 2017 to March 2017. Ondo West Local Government is located on Latitude 7.1° North and Longitude 4.83° East and 277 m above the sea level. It is primarily inhabited by the Yoruba ethnic group though there are other ethnic groups such as Igbo and Hausa. The two major religions are Islam and Christianity. The town consists majorly of civil servants, businessmen, artisans and students of various institutions such as Federal College of Education, State University (University of Medical Sciences) and Private University (Wesley). There are five major government health facilities in Ondo West LGA namely; Mother and Child Hospital, Trauma and Surgical Centre, Kidney Care Centre, State Specialist Hospital and Forty-four Primary Health Care (PHCs) centres.

#### Participants

All teenage mothers in Ondo West LGA with at least one under-five child was eligible for the study.

#### Sample size determination and sampling technique

The sample size was calculated using Leslie Fischer’s formula for a single population proportion [n = Z^2^p(1 − p)/d^2^]. The prevalence of malnutrition among under five was 23.6% from a previous study [12], with a 95% CI and precision of 5%. After accounting for a non-response rate of 10%, the total sample size was rounded up to 300. Multi-stage sampling technique was used in selecting participants. In the first stage, out of twelve wards, seven were selected by simple random sampling technique (balloting method). In the second stage, twenty-two PHCs were purposively selected from the forty-four available PHCs in the selected wards based on locations, sizes and services offered. The third stage involve the selection of teenage mothers with at least one under-five child attending the centres to access any service by simple random sampling technique.

#### Data collection tools and technique

A structured interviewer-administered questionnaire was used to collect data. The questionnaire had four sections which assessed the respondents’ socio-demographic characteristics, Index child characteristics, breastfeeding practices and nutritional status of the index child using anthropometric measurements. The questionnaire was pretested among teenage mothers attending General Out-patient Clinic and Immunization Clinic at State Specialist Hospital, Ondo. The instrument was translated into the local language (Yoruba) and back-translated to English language. Five research assistants were recruited and trained for 2 days to assist with data collection. Breastfeeding practices of teenage mothers were determined by assessing the time of initiation of breastfeeding and the duration of exclusive breastfeeding. Nutritional status of the under-five children was generated based on the output from the World Health Organization (WHO) Anthro software. WHO standard classification was used to categorize the nutritional status of the under-five children [11].

#### Data analysis

Data collected were analyzed using the Statistical Package for Service Solution (SPSS version 20). Socio demographic, breastfeeding practices and nutritional status of participants were presented in texts and tables. Bivariate analysis was done to determine the association between teenage mothers’ breastfeeding practices and the nutritional indices of the under-five children, and variables with p < 0.2 were included in the multivariate logistic regression model. In the multivariate analysis, predictors with p < 0.05 were considered statistically significant.

### Results

#### Socio-demographic characteristics of the study participants

The sample consisted of 300 mother–child pair who started the survey with 285 completing it. This gave a response rate of 95%. Most (84.2%) of the mothers are aged 18–19 years, 54.7% delivered their first child at age 17 years and 90.5% of the mothers have one under-five child. Majority (81.1%) are of Yoruba tribe and practice Islam (48.4%). Forty per cent (40%) had primary education and are mostly traders (77.5%). About 93.7% of the respondent household income per month was more than 20,000 naira. All respondents breastfed their children. Majority (87%) initiated breastfeeding in less than 1 h while 31.9% breastfeed their children exclusively for 6 months (Table 1).

**Table 1:** Socio-demographic characteristics’ of teenage mothers

Variables	Frequency (n = 285)	Percentage (%)
*Age (years)*		
15–17	45	15.8
18–19	240	84.2
*Age at delivery of first child*		
15	27	9.5
16	102	35.8
17	156	54.7
*Number of under-five children*		
One	258	90.5
Two	27	9.5
*Ethnicity*		
Hausa	18	6.3
Igbo	36	12.6
Yoruba	231	81.1
*Religion*		
Christianity	128	44.9
Islam	138	48.4
Traditional	19	6.7
*Level of education*		
None	25	8.8
Primary	114	40.0
Secondary	74	26.0
Tertiary	72	25.2
*Occupation*		
Full housewife	9	2.8
Trading	221	77.5
Artisan	18	6.7
Farming	37	13.0
*Household income/month*		
< 20,000	18	6.3
> 20,000	267	93.7
*Breastfeeding*		
Did you breastfeed your child?		
Yes	285	100
*Initiation of breastfeeding (h)*		
< 1	248	87.0
> 1	37	13.0
*Duration of exclusive BF (months)*		
< 4	45	15.8
4–5	149	52.3
6	91	31.9

#### Nutritional status of under-five children of teenage mothers

The mean age of the children was 32.9 (± 5) months. The average weights and heights were 14.67 (± 2) kg and 67.36 (± 4) cm respectively. Majority (73.4%) of the children were undernourished, almost 10% were over-nourished and less than 20% were having normal nutritional status. Among the undernourished, the prevalence of underweight was the highest (29.5%), followed by wasting (25.3%) and stunting (18.6%) (Table 2).

**Table 2:** Anthropometry and nutritional status of under-five children of teenage mothers *(n *=* 285)*

Variables	Frequency (n)	Percentage (%)
*Age (months)*		
6–17	74	26.0
18–35	64	22.5
36–60	147	51.5
Mean = 32.9 ± 5		
*Weight (kg)*		
10–12	46	16.1
13–15	120	42.1
16–18	119	41.8
Mean = 14.67 ± 2		
*Height (cm)*		
≤ 50	46	16.1
51–60	46	16.1
61–70	130	45.7
≥ 71	63	22.1
Mean = 67.36 ± 4		
*Nutritional status*		
Normal	48	16.8
Over-nutrition	28	9.8
Under-nutrition	209	73.4
*Undernutrition		
Stunted	53	18.6
Wasted	72	25.3
Underweight	84	29.5

*The proportions of undernutrition totaled 73.4% (209/285)

#### Relationship between breastfeeding practices and nutritional status

There are statistically significant relationships between breastfeeding initiation time and occurrence of stunting ($\chi^2$ = 7.095, p = 0.008), and underweight ($\chi^2$ = 29.684, p = 0.001) in under-five children. Duration of breastfeeding was found to be statistically significant to the occurrence of wasting ($\chi^2$ = 6.910, p = 0.009) in under-fives. Logistic regression further showed that teenage mothers who breastfed after 1-h of birth are ten times more likely to have stunted children and almost seven times more likely to have underweight children compared to those who breastfed early. Also, children born to mothers who practised exclusive breastfeeding for less than 6 months are two times more likely to be wasted compared to those who exclusively breastfed for 6 months (Table 3).

**Table 3:** Relationship between breastfeeding practices and nutritional indices of under-five children of teenage mothers (n = 285)

Variables	Height for age		Total	$\chi^2$	p-value
	Stunting	Normal			
BF Initiation					
≤ 1 h	52 (21.0)	196 (79.0)	248 (100)	7.095	0.008*
> 1 h	1 (2.7)	36 (97.3)	37 (100)		
BF Duration					
< 6 months	37 (19.1)	157 (80.9)	194 (100)	0.091	0.763
6 months	16 (17.6)	75 (82.4)	91 (100)		

Variables	Weight for age		Total	X^2^	p-value
	Underweight	Normal			
BF Initiation					
≤ 1 h	59 (23.8)	189 (76.2)	248 (100)	29.684	0.001*
> 1 h	25 (67.6)	12 (32.4)	37 (100)		
BF Duration					
< 6 months	56 (28.9)	138 (71.1)	194 (100)	0.108	0.742
6 months	28 (30.8)	63 (69.2)	91 (100)		

Variables	Weight for height		Total	X^2^	p-value
	Wasted	Normal			
BF Initiation					
≤ 1 h	63 (25.4)	185 (74.6)	248 (100)	0.020	0.888
> 1 h	9 (24.3)	28 (75.7)	37 (100)		
BF Duration					
< 6 months	58 (29.9)	136 (70.1)	194 (100)	6.910	0.009*
6 months	14 (15.4)	77 (84.6)	91 (100)		

Variables	Odds ratio (OR)	Confidence interval	p-value
*Predictors of nutritional indices of under-fives*			
***Predictor of stunting***			
Breastfeeding initiation time			
> 1 h	9.551	1.279–16.310	0.028*
≤ 1 h (Ref)	1		
***Predictor of underweight***			
Breastfeeding initiation time			
> 1 h	6.674	3.159–14.097	0.001*
≤ 1 h (Ref)	1		
***Predictor of wasting***			
Duration of exclusive BF			
6 months	2.346	1.228–4.480	0.009*
< 6 months (Ref)	1		

*BF* breastfeeding

*Significance

### Discussion

All the respondents breastfed their under-five children at different times after birth. This agrees with a study in Salisbury where more mothers also breastfed after birth [13]. The result of this study was much higher than the report of 35% of mothers who decided to breastfeed in a previous study [9]. Similarly, it was above 38% breastfeeding prevalence in another study [14]. However, our finding agrees with the 80% and 90% reported by Smith et al. [15] and Chatman et al. [16] respectively in their previous study. The high prevalence of breastfeeding reported in this study might have resulted from constant health talks that they are exposed to during ante-natal clinics and the free medical care initiated by the State [17].

Eighty-seven per cent of our respondents initiated breastfeeding within 1 h after birth. This was much higher than what was previously reported by the State in 2013 [2] and even higher than 35.1% reported by Park et al. [18] in their study among teenage mothers. The finding agrees with 80% reported by Chatman et al. [16] in a previous study among Jamaican mothers.

For optimal growth, it is recommended that infants be exclusively breastfed for the first 6 months of life [19]. Ninety-one (31.9%) of the respondents breastfed their children exclusively for 6 months. This finding was higher than the report in previous studies among older mothers in Nasarawa, Ondo and Osun States, where the prevalence of exclusive breastfeeding was 18.1%, 16.7% and 16.4% respectively [20–22]. However, the finding agreed with the prevalence of 30.1% of exclusive breastfeeding in a study conducted in Zambia [23]. The high prevalence of exclusive breastfeeding may have been influenced by constant nutrition education and health benefits of breast milk that they are exposed to during ante-natal clinics. Unfortunately, it was observed that majority of the participants could not sustain their desire to exclusively breastfed for 6 months.

The prevalence of malnutrition among under-five children born to teenage mothers was very high. Four out of five (83.1%) of these children are malnourished. The most prevalent form of malnutrition among them was under-nutrition (73.4%). This finding agreed with the findings of other researchers [24, 25] showing that malnutrition is indeed a teething public health problem among under-fives. The prevalence of underweight, wasting and stunting in this study was higher than the prevalence of underweight (25%), wasting (9.4%) and stunting (19.2%) among under-fives in a study in Bangladesh [26]. The prevalence reported in the present study was low compared to the prevalence of underweight (33.3%), wasting (26.4%) and stunting (24.6%) reported in a previous study [24]. The findings support the view that the prevalence of malnutrition among under-five children is still high in many developing countries [12]. The problem is further compounded by teenage motherhood who are not yet adequately prepared to shoulder the responsibility of childcare which is one of the causes of childhood malnutrition [27].

The association between breastfeeding practices and nutritional indices of under-five children showed that early initiation of breastfeeding was associated with a lower prevalence of stunting and underweight among under-five children. This agreed with the report that better child feeding practices were associated with higher HAZ among 12- to 36-month-old children in research conducted by Ruel and Purnima [28] among Latin Americans. Also, this study showed that breastfeeding exclusively for 6 months is associated with lower prevalence of wasting among under-fives. This agreed with the finding of another researcher [24] who concluded that children who did not receive appropriate feeding had higher odds for wasting, stunting and underweight.

### Conclusion

This study shows that undernutrition is the most prevalent form of malnutrition among the under-fives of teenage mothers. Although many initiated breastfeeding on time, but could not sustain the practice for 6 months. Also, the risk of stunting and underweight increases with late initiation of breastfeeding at birth while the risk of wasting increases with exclusive breastfeeding duration less than 6 months. Therefore, education of teenagers on the consequences of early motherhood and helping sexually active teenagers in meeting their contraceptive needs and uptake will help to curb the problem of teenage pregnancy and reduce the high prevalence of under-five malnutrition.

### Limitation of the study

The study sample size is small which may affect the generalizability of results, and there is a likelihood of a recall bias as responses were self-reported.

## Abbreviations

BMI: Body Mass Index
IYCF: infant and young child feeding
NDHS: Nigeria Demographic and Health Survey
PHCs: Primary Health Care
SD: standard deviation
WHO: World Health Organization
